# Professionalizing public health education in the Eastern Mediterranean Region

**DOI:** 10.3389/fpubh.2025.1656182

**Published:** 2025-09-12

**Authors:** Elsheikh Badr, Sara Ali, Zainah Assaf, Abdelmounim Belalia, Iman Nuwayhid, John Middleton, Katarzyna Czabanowska, Neil Squires, Rajaa Al-Raddadi, Yousef Khader

**Affiliations:** ^1^Center for Health Workforce Development, RAK Medical and Health Sciences University, Ras Al-Khaimah, United Arab Emirates; ^2^GHD|EMPHNET, Amman, Jordan; ^3^Universite Mundiapolis, Casablanca, Morocco; ^4^Faculty of Health Sciences, American University of Beirut (AUB), Beirut, Lebanon; ^5^University of Wolverhampton, Wolverhampton, United Kingdom; ^6^Department of International Health, Care and Public Health Research Institute (CAPHRI), FHML, Maastricht University, Maastricht, Netherlands; ^7^Department of Health Policy Management, Institute of Public Health, Jagiellonian University, Krakow, Poland; ^8^UK Health Security Agency, London, United Kingdom; ^9^King Abdulaziz University, Jeddah, Saudi Arabia; ^10^Department of Community Medicine, Public Health and Family Medicine, Faculty of Medicine, Jordan University of Science and Technology, Irbid, Jordan

**Keywords:** public health, health workforce, professional education, competency framework, Eastern Mediterranean Region, public health professional education, EMPHNET, public health credentialing/credentials

## Abstract

During the eighth EMPHNET Regional Conference, a roundtable session gathered eight public health leaders and academics from the Eastern Mediterranean Region (EMR) and Europe to discuss the professionalization of public health education with a focus on the EMR. The discussion reviewed the achievements, challenges, and prospects of public health education in the EMR and identified the tenets and strategies for professionalizing public health education and the public health workforce to better address health systems challenges. It also introduced the best practices and innovative approaches of public health professional education, and proposed strategies and interventions to strengthen public health education in the EMR toward professional and competency-based approaches. The findings highlighted that public health is not seen as an attractive career in the EMR due to the lack of clear career pathways and limited recognition. This has significant implications for education in the region, emphasizing the need to professionalize the public health workforce by leveraging international and regional experiences to address local challenges. The findings also underscored the importance of adopting competency-based approaches and pursuing professional recognition, credentialing, and regulation. In response, panelists recommended mapping and reviewing existing educational programs to develop competency-based frameworks and curricula tailored to the region’s context. They also stressed the importance of fostering partnerships between academia and public health organizations to provide experiential training and education in public health.

## Introduction

The Eastern Mediterranean Region (EMR) is experiencing a shortage of qualified health workers. Overall, health worker production and availability are suboptimal and unbalanced across the region (1). In response to these challenges, the WHO Regional Office for the Eastern Mediterranean developed a Framework for Action for Health Workforce Development to improve the capacity of countries to plan and manage their health workforces sustainably and effectively (2). Within this context, the public health workforce in the EMR faces the additional challenge of undermining. Public health is not an attractive career in the region due to a lack of credible and recognized educational qualifications, inadequate recognition, and a lack of job structure and career prospects. The medical and clinical dominance make it more appealing and rewarding for the candidates to pursue careers in these disciplines. The COVID-19 pandemic has exposed both the health workforce crisis and the need for the public health workforce in the region.

Public health education is rooted in the EMR, and various institutions provide basic and graduate public health education. There are, however, concerns about the quantity, quality, and relevance of graduates of public health programs in the region. A review covering the 22 Arab Countries, which form the majority of the EMR, found that the capacity for education is limited. Notably, 10 countries lack any MPH program (3). The educational orientation for public health is mainly academic, with minimal to no adoption of practice-based training models apart from some initiatives in certain fields. Therefore, calls are emerging in the region to scale up competency-based public health education (4).

Demands for a high level of professionalism in public health practice, as well as the corresponding strengthening of public health education to meet 21st-century community challenges, present an opportunity to develop a new generation of public health professionals who are capable of meeting the needs of local populations. To address these demands, public health education must transition from being simply ‘process-oriented, focusing on knowledge and skills, to becoming ‘competency-based and outcome-oriented’. This approach emphasizes the synthesis of knowledge and skills across multiple contexts. It focuses on the development of the professional competencies necessary for effective public health practice. In that way, public health education can contribute effectively to professionalizing the public health workforce. This is considered essential to strengthening public health for a new era (5).

This viewpoint explores the status of public health education in the EMR and highlights the critical needs for its professionalization. It draws insights from a distinguished panel of eight public health leaders and academics from the EMR and Europe, who participated in a roundtable discussion during the eighth EMPHNET Regional Conference. The panelists came from certain countries in the EMR, including Jordan, Lebanon, Morocco, Saudi Arabia, and Sudan. However, their expertise spans the entire region, as most of them are involved in regional initiatives or roles related to public health education, practice, and research. Three panelists came from outside the EMR, from the Netherlands and the UK. They are leaders in public health who are heavily involved in European and global public health initiatives, including competency frameworks and the professionalization of the public health workforce.

The panelists and roundtable participants discussed the challenges and opportunities for advancing public health education and fostering its professionalization, with a particular focus on the EMR. The roundtable brought together 35 public health professionals from diverse countries and organizations, including ministerial officials, policymakers, academics, educators, regional focal points, and Field Epidemiology Training Programs (FETPs) residents and graduates. This diverse group contributed to a rich exchange of perspectives and ideas.

## Professionalization of the public health workforce: implications for education in the EMR

The professionalization of the public health workforce is promoted globally as a model that advocates for the development of competency frameworks for outcome-based education, as well as the strengthening of the workforce through formal organization, professional credentialing, and codes of ethics (6). Embracing this professionalization agenda in the EMR is a critical demand to address the emerging health challenges, resulting from pandemics and other complex issues faced by several countries in the region. In light of this ambitious reform agenda, the roundtable moderator highlighted the importance of enhancing the public health capacity in the EMR. He emphasized the fact that the region lacks a well-prepared public health workforce and professionals to respond to the challenging health landscape resulting from pandemics like COVID-19 and the difficult situations faced by some countries in the region.

One of the panelists shed light on four aspects that should be considered when answering the question: “How can the public health stakeholders in the EMR best advance the public health profession?” These aspects are the context of the region, the quest to professionalize the public health workforce, the implications for public health education, and the global development and its impact on the region. The panelist expressed the need for defining the competencies, educational standards, credentialing, and code of ethics to professionalize public health.

To address these needs, establishing a common understanding of the definition of public health education and the composition of public health is an essential step toward professionalizing public health education (7). One of the panelists argued for the endorsement of the Master of Public Health degree as a primary degree, advocating for comprehensive and interdisciplinary public health education (3). And other panelists support a multidisciplinary approach to public health, acknowledging that effective public health practice requires competence from diverse professions such as medicine, nursing, environmental health, and social sciences (8).

Furthermore, Winslow’s traditional definition of public health (9), “the science and art of promoting health, preventing disease, and prolonging life through the organized efforts of society,” was emphasized. This concept sheds light on the societal dimension of public health, extending beyond the hospital and healthcare system to encompass the wider determinants of health. As such, the public health workforce comprises both core public health professionals employed in specialized agencies and a broader workforce whose activities substantially influence population health, including healthcare providers, educators, social workers, and policymakers.

Public health education is essential for training the necessary core public health professionals and supplementing the knowledge of students in clinical disciplines. One panelist highlighted that public health education should reflect the needs of the region; that can be accomplished by aligning the educational standards with the available competency frameworks and roadmaps. She referred to examples such as the WHO’s Roadmap for Emergency Workforce, the Association of Schools of Public Health in the European Region (ASPHER) framework, in addition to discussing existing competency frameworks, such as the WHO Global Competency and Outcome Framework, and the ECDC’s core competencies in applied infectious disease epidemiology.

## Linking global perspectives to the EMR

Benefiting from international experiences and initiatives is a cornerstone in advancing the professionalization of public health education that the countries of the EMR should consider. One panelist discussed the UK’s multidisciplinary approach to public health training, which includes recruiting from diverse disciplines. The training is competency-based and spans 4–5 years, aiming at enabling the workforce to focus on health protection, health improvement, and healthcare-related public health, and providing them with the needed set of competencies. He shed light on the interrelationships among the competency domains that the UK Faculty of Public Health highlights, emphasizing that the dimension of healthcare-related public health is not included in public health programs worldwide.

The UK’s public health training, which has an evident focus on experiential learning and a practice-based approach to acquiring competence, is an example to emulate in many settings, including the EMR (10).

Another panelist emphasized the relevance of international public health training models to the region. The panelist focused on the need to leverage foundational knowledge gained through basic training programs and university education. This knowledge is essential to address workplace challenges and navigate the complexities unique to the region. He also underscored the critical need for strong public health leadership capable of mobilizing resources and organizing multidisciplinary teams to work toward shared goals and missions. Additionally, he acknowledged the regional role of the WHO in developing specific competencies to address the region’s unique needs. This ensures the effective delivery of the renewed set of essential public health functions in the EMR (11).

## Professionalizing public health education in the EMR: experiences and reform agenda

Public health education has witnessed significant growth in the EMR; however, persisting challenges still occur (3). One of the panelists showcased the growth in Master of Public Health (MPH) programs in the Arab countries between 1960 and 2019, followed by a summary of the mapped graduate public health programs across the region. Although this is a remarkable progress, many countries still lack a well-developed public health education infrastructure, which necessitates the need for improved standards and the adoption of competency-based education. Assessing the quality of these programs is challenging, with statistics showing that 53% of the faculties of public health offer MPHs that are fully theoretical without hands-on or practical experience compared to the faculties of medicine. The panelist also emphasized the importance of aligning both the public health education and the health system. And he stressed the need to build a common vision for public health education in the region, recommending that this vision should transform the education system into tools to be utilized in empowering and strengthening the public health system.

One of the panelists presented the Saudi experience in community medicine, also known as preventive medicine. She pointed out that this training is confined to physicians and includes clinical medicine and public health. The structure of the preventive medicine residency program in Saudi Arabia includes academic coursework, public health practice rotations, and clinical training. This program represents a structured approach to developing essential competencies in public health. It allows the public health workforce and professionals to work in the intersections of the healthcare system in the Kingdom, enabling them to apply their expertise to clinical situations and work in clinical practice at the population level. Another panelist shared the experience of Field Epidemiology Training Programs (FETPs) in the region, highlighting them as hands-on, competency-based educational models that prepare field epidemiologists through practice-oriented training. Unlike the preventive medicine program, FETPs enroll candidates from diverse disciplinary backgrounds (12).

Moving to the western and francophone part of the Arab World, one speaker on the panel emphasized the urgent and increasing need for public health education and training. He pointed out that the COVID-19 pandemic was a great testimony. The National School of Public Health, in Morocco, was mentioned as an example. It adopts competency-based education, aiming at preparing public health professionals across four dimensions of competence: scientific, technical, managerial, and leadership. The panelist explained that the effectiveness of adopting the competency-based education lies in offering learners engaging and interactive learning experiences, utilizing the integration of more interdisciplinary competencies such as public health ethics, systems thinking, problem-solving, and equity.

In this context, adopting a competency-based approach that focuses on building, empowering, and assessing the essential knowledge, skills, and attitudes should be considered when professionalizing public health education (13). All of the roundtable speakers emphasized the importance of reviewing and aligning existing competencies in the region with the essential public health functions. This ensures that public health graduates are prepared with the necessary competence to address healthcare concerns and are assessed through workplace-based approaches rather than summative theoretical exams. This underscores the importance of having a guiding framework of core competencies to inform pedagogical and assessment methods, promote effective learning and program evaluation, and establish a pathway for adopting a comprehensive, practice-based educational approach in public health. The agenda for transforming public health education in the EMR can be summarized based on the roundtable discussion in specific imperatives that include: developing competency frameworks, introducing competency-based curricula, promoting practice-based learning strategies, adopting work-based assessment, and evaluating programs for effectiveness.

## Toward a framework for core competencies

At the international level, the United Nations High-Level Commission on Health Employment and Economic Growth (the Commission) proposed 10 recommendations, including five immediate ones. These recommendations emphasize expanding transformative, high-quality education, as well as shifting education models away from limited specializations and toward lifelong learning and developing regionally relevant skills (14). Two panelists emphasized the importance of competency frameworks as tools for defining and assessing essential competencies and enabling experiential learning. Also, they highlighted that existing competency frameworks developed by organizations like the WHO, ASPHER, and the UK Faculty of Public Health can serve as models for deriving and developing competency frameworks that are more related to the region.

Also, the World Health Assembly resolution on the Global Strategy on Human Resources for Health: Workforce 2030 urged professional councils, associations, and regulatory bodies to implement regulations to optimize workforce competencies and support interprofessional collaboration for a skill mix responsive to population needs (15).

At the regional level, in response to these pressing needs, the International Academy of Public Health (IAPH), based in Amman, is pioneering efforts to advance public health education and professionalize the workforce in the region and beyond. A significant step in this initiative is IAPH’s development of a competency framework tailored for public health education in the region, setting essential standards for the knowledge, skills, and attitudes required to meet regional health challenges. The envisioned framework emphasizes the concept of entrustment and establishes a set of entrustable professional activities (EPAs) as units of practice to guide curricula and educational programs (16). In the IAPH model, EPAs are organized around core competency domains specific to public health functions, such as assessment, promotion, prevention, and protection, as well as generic competencies, including communication, leadership, professionalism, and agility. The IAPH competency framework has the potential to support the transformation of public health education in the EMR toward practice-based models. All panelists welcomed this initiative and called for expediting efforts to finalize and publicize the IAPH competency framework.

## Professional recognition, credentialing, and regulation

There is a wide range of professionals in the public health workforce, and every country has a different approach to providing public health services. The structural challenges of the public health workforce supply and demand in different organizations and health systems are also reflected in the range and complexity of the public health workforce’s professions. Therefore, to address public health concerns, a qualified and responsive public health workforce must be credentialed, regulated, and formally recognized (17). As compared to the medical and clinical disciplines, the public health profession is the least regulated in many settings globally and across the EMR (18).

Two members of the panel emphasized the importance of developing professional standards and accreditation mechanisms. They pointed out the significant role of organizations such as ASPHER and the Agency for Public Health Education Accreditation (APHEA) in establishing standards and accrediting public health programs across Europe. Another panelist highlighted the absence of strong accreditation systems for public health education in numerous countries in the EMR and emphasized the necessity for regional and national accreditation initiatives (3). It is essential to establish professional bodies for public health practitioners to ensure accountability, promote ethical conduct, and safeguard public interest. It is worth mentioning that the role of these professional bodies, such as chambers and associations, in providing assurance and enforcing codes of conduct was emphasized. The discussion raised issues regarding the licensing of public health professionals in the region. Academic public health qualifications, such as a Master of Public Health (MPH), are not widely recognized by licensing bodies. Apart from community/preventive medicine residency education programs, other public health educational qualifications are not eligible for professional licensing. The professionalization agenda discussed at this roundtable has the potential to transform public health education into credible, practice-based models that allow for full professional licensure of a multidisciplinary public health workforce.

Advocating for public health and securing investments are essential to professionalizing the workforce. One of the panelists speaks to the need for clear vision and leadership in public health, underscoring the importance of articulating a compelling investment case. Another panelist calls for smarter advocacy strategies to improve health outcomes by increasing workforce capacity. This includes enhanced disease surveillance and effective emergency response. Building strong partnerships across sectors, such as education, finance, and social welfare, is essential to strengthening the case for public health investment. These partnerships contribute to increased support for workforce development initiatives.

## Addressing contextual challenges in the EMR

The EMR is a vibrant and diversified region with challenging social, economic, political, and demographic realities. The average life expectancy among the 679 million people inhabiting the region varies from 55.4 years in Somalia to 80.7 years in Qatar (19, 20). Some countries have very low poverty rates, while others have more than one-third of the population living below the international poverty line (17). Despite the financial prosperity of some countries, the region confronts significant health concerns. Health policies are typically inadequate, health systems are under-resourced and poorly managed, and humanitarian situations caused by conflicts are posing significant challenges in the region (21).

Professionalizing public health education in the region necessitates overcoming regional problems. One member of the panel emphasized how political instability and wars affected health and education systems, interrupting capacity-building efforts. Another member highlighted the importance of knowing the geopolitical backdrop influencing health and well-being. The diversity of public health systems across the region requires tailored approaches. A third panelist demonstrated this with changes to Saudi Arabia’s preventive medical training, whereas a fourth panelist focused on existing regional assets to increase capacity. Additionally, collaboration and knowledge sharing are crucial, as highlighted by the advocacy by participants for a regional academic network.

## Conclusion and recommendations

Improving public health education in the EMR requires a tailored approach that addresses the unique challenges of the region while leveraging its existing resources. By implementing competency-based training programs, establishing systems for recognizing and regulating standards, fostering collaboration within the region, and advocating for greater investment in healthcare workers, countries in the region can cultivate a skilled public health workforce. This workforce will be equipped to address the complex healthcare challenges of the countries in the region and contribute to a healthier future for all. Scaling up the public health workforce capacity in the region requires professionalizing public health education by implementing specific reforms. These reforms should ideally include introducing a competency framework, developing outcome-based curricula, aligning pedagogy and assessment with the required competencies, evaluating programs for effectiveness and impact, and illuminating pathways for countries to adopt contextualized models of practice-based education.

To move forward, it is recommended to map and cross-reference existing educational programs at the international, regional, and national levels in order to establish educational standards that are tailored to the needs of the region. It is essential to engage stakeholders in defining competencies and developing job descriptions for the public health workforce to create clear career pathways and define the terms of responsibilities. Additionally, fostering partnerships between academic institutions and public health organizations is crucial to providing experiential learning opportunities that enable the practical application of public health training and education. In this context, countries and entities in the EMR are encouraged to adopt a transformative agenda based on a professionalization model for public health education to ensure the development of a public health workforce that is fit for purpose and fit for practice, leading to a healthier and more prosperous population.

## Data Availability

The original contributions presented in the study are included in the article/supplementary material, further inquiries can be directed to the corresponding author.
